# Patterns of smoking and its association with psychosocial work conditions among blue-collar and service employees of hospitality venues in Shenyang, PR China

**DOI:** 10.1186/1471-2458-10-37

**Published:** 2010-01-27

**Authors:** Xun Li, Huiying Liang, Xuelian Li, Peng Guan, Zhihua Yin, Baosen Zhou

**Affiliations:** 1 Department of Epidemiology, China Medical University, Shenyang, PR China; 2 Shenyang Center for Disease control and Prevention, Shenyang, PR China

## Abstract

**Background:**

To characterize the smoking patterns of hospitality employees in blue-collar and service occupations, and to examine its relations with psychosocial work conditions.

**Methods:**

The Shenyang Hospitality Industry Employees Survey-a face-to-face cross-sectional study of representative hospitality industry employees-was conducted between March and July 2008. A total of 4,213 workers were selected using stratified random cluster sampling designs, and final analyses were performed on 2,508 blue-collar and service subjects. Multilevel-logistic regression models were used to estimate the contribution of psychosocial work conditions to smoking status.

**Results:**

Blue-collar and service employees smoked at a rate 1.4 times that of the general population (49.4% *vs. *35.8%), more particularly for females (12.9% *vs. *3.08%). Strain jobs had significantly higher odds ratio of daily smoking (OR 2.09, 95%CI: 1.28-3.41) compared to the relaxed category. The passive jobs (OR 2.01, 95%CI 1.27 to 3.17), highest job demands (OR 1.72, 95%CI: 1.13-2.61), and lowest job control (OR 2.56, 95%CI: 1.57-4.16) were also associated with a significantly higher daily smoking ratio. The negative relationship between job stability and smoking behavior was slightly stronger among daily than occasional smokers. However, neither job strain nor any of its components was found to be significantly associated with occasional smoking.

**Conclusions:**

Smoking in hospitality blue-collar and service employees is certainly a major occupational health problem in Shenyang. This evidence also suggests an association between psychosocial-work conditions and smoking status, and implies that more intervention studies where changes in work environment are carried out in combination with health promotion interventions should be performed.

## Background

There is extensive epidemiological evidence of increased mental and physical illness morbidity and mortality in populations exposed to adverse psychosocial work environments [1-3]. For example, previous studies have linked job strain to hypertension, cardiovascular disease, psychosomatic symptoms, depression, adverse birth outcomes, lung cancer, and other respiratory disorders among different occupational status [3-7]. Although the precise pathways and biological mechanisms underlying these associations have not yet to be established, according to modern occupational strain theories, two potential separate mechanisms are worthy of consideration [8-12]. First, acting directly via the organism's main strain axes, occupational strain can affect the psycho-physiological responses (automatic, neuroendocrine, and immune) that are implicated in pathology and host vulnerability, reducing resistance to pathogens, or directly stimulating disease mechanisms. Second, acting indirectly via unhealthy behaviors (e.g., smoking, alcohol consumption), which may be used either deliberately or inadvertently as a coping mechanism to deal with stress [13]; almost all smokers attribute their smoking at least partly to its alleged calming and relaxing properties [14]. In order to trace such intermediate mechanisms for highly prevalent diseases, such as blood pressure [15], coronary heart disease [16], and lung cancer [7], some studies have focused on the association of job strain with their major risk factor, smoking [17,18].

However, previous empirical research, which predominately analyzed small samples, used inconsistent measures of job strain components, and did not differentiate between occasional and daily smokers, has produced mixed results on the association between psychosocial work environment and smoking. Employees exposed to adverse psychosocial work characteristics showed a higher prevalence and/or intensity of smoking in some studies (occupational stress [8,19], high job demands [20,21], low job control [9,22], or job strain [9,23]), but other studies didn't find these associations [24,25]. Furthermore, Tsutsumi *et al. *[20] have shown a smaller quantity of cigarettes smoked to be associated with low job control; and in another study [26] high job strain was associated with a lower prevalence of smoking.

In the field of occupational health in China, the main concern is with exposure to occupational hazards [27]; smoking is often not a priority for health promotion or protection in the workplace. There are few reports which focus specifically on the relationship between psychosocial work conditions and patterns of smoking in hospitality industry personnel, and none of these focused on blue-collar and service employees. Working in the hospitality industry (hotels, motels, restaurants, bars, casinos, coffee shops, and karaoke lounges) is generally regarded as a stressful occupation. Apart from receiving stressors that are common to most other workplaces, the employees are also exposed to stressors that are specific to the hospitality settings. Generally, hospitality jobs are considered to be a precarious job, with low pay and low procedural justice (decision-making procedures), lack of esteem, lack of job autonomy, lack of control over working conditions, and a lack of promotional prospects [28-33]. Blue-collar and service employees of hospitality venues are also exposed to physical stressors (e.g., noise, secondhand smoke, long working hours, and shift work) and psychosocial stressors cover job characteristics (e.g., work load, variety, clarity) [34-36].

Due to a relatively high turnover among blue-collar and service employees, it is difficult to investigate, and thus relatively few studies have been carried out. The lack of exact knowledge regarding the patterns of smoking among Chinese hospitality blue-collar and service workers and its mixed relationship to psychosocial working environment provided the rationale for the present study. We aimed to explore the patterns of smoking and its association with psychosocial working conditions among blue-collar and service workers employed in hospitality venues in Shenyang, China.

## Methods

### Sample and data collection

The Shenyang Hospitality Industry Employees Survey (SHIES), a cross-sectional study conducted between March and July 2008, designed to explore associations between the psychosocial work environments of hospitality venue employees and their smoking behavior, health status, and sickness absence.

The SHIES used a stratified random cluster sampling design, in which all venue units (including hotels, motels, casinos, coffee shops, karaoke lounges, restaurants, bars, nightclubs, and cabaret) were first divided into three strata (upscale, mid-level, and low-level) on the basis of their registered capitals. Within each stratum, a certain number of venues were randomly selected, with probability of selection proportional to the population size of the units. Total sample size of SHIES is 60. The number of the sample units allocated to the three strata was decided by weighted mean (upscale: 17, mid-level: 19, and low-level: 24). Then cluster sampling was conducted on the selected venues.

The present study was approved by the Institutional Review Board of the China Medical Board. After obtaining informed consent, face-to-face interviews, lasting an average of 31 minutes, were conducted for all subjects by trained interviewers using a structured questionnaire. Detailed information was collected on demographic characteristics, psychosocial work conditions, smoking behaviors, interpersonal factors, health status, work-related injuries, and sickness absence. The same interview protocol was used across each venue to ensure identical interview and data collection procedures. To guarantee the reliability of responses, we also checked all questionnaires for missing data and followed up to obtain the relevant information.

Of the population of 4213 employees, 3896 consented to participate in the research (participation rate 92.5%). Of these, 2508 employees in blue-collar and service occupations 15 years or older who had lived in this venues for at least one year (1433men and 1075women) completed all the relevant questions in the questionnaire and were included in the final analyses.

### Measures of smoking behavior

Information regarding current smoking, frequency, number smoked, and smoking history was assessed through self-report. Never-smokers were those who had never smoked or smoked less than 100 cigarettes during their lifetime. A current smoker was defined as someone who had smoked 100 or more cigarettes in his/her lifetime and reported smoking within 30 days before survey was conducted, daily smokers were defined as those who smoked every day, and occasional smokers were defined as those who smoked on some days within the past month. An ex-smoker was those who smoked at least 100 cigarettes in their lifetime, but were not smoking for the preceding one month.

### Work related psychosocial factors

#### Job Strain

Assessment of job strain was based on a modified job content questionnaire comprised of the job demand scale (Cronbach α = 0.76) and job control scale (Cronbach α = 0.82) [37,38]. Three questions addressed the psychological demands of the job, that is, having high workload and working at a high pace and not having enough time to complete work tasks. Job control was assessed with nine questions about the worker's ability to use and develop skills and exert decision authority. The responses were given on a Likert scale of 1 = "very little" to 5 = "very much". A total score for both constructs was computed and the scores were further divided into quartiles to indicate different exposure levels. To create a job strain indicator, demands and control were split on the median and combined to four categories: relaxed jobs (low demands combined with high control), active jobs (high demands combined with high control), passive jobs (low demands combined with low control), and strain jobs (high demands combined with low control) [39,40].

#### Job stability

The job stability was also investigated in this study as a separate category, because it could be regarded as exposed to even higher levels of anxiety and, more important, insecurity than the job-strain category. For the stability of the job, subjects were asked "How do you think about the stability of your current work?", with answers: absolutely stable, maybe stable, maybe unstable, absolutely unstable. In analysis, these who chose "absolutely stable" are identified as "stable", on the contrary those who chose "maybe unstable" or "absolutely unstable" are identified as "unstable".

### Measures of background covariates

#### Demographic variables

Information regarding age, gender, ethnicity, level of education, marital status, total years of working within the hospitality settings, and per month income/household was obtained through self-report.

#### Interpersonal factors

There also was a serious of questions on the smoking status of parents with whom the respondents were living, as well as the smoking status of peers. Respondents were said to have positive peer influence if more than one of their close friends were smokers.

#### Attitude and belief factors

These effects were measured by the statement: "Smoking is an easy way to enjoy yourself.", with two-point scale "agree" or "disagree".

### Data analysis

Patterns of smoking were expressed as the percentage of subjects found non-smoking, occasionally smoking, and daily smoking. Comparisons between proportions were performed using an adjusted chi-squared test for clustered data [41]. Odds ratios (ORs) and 95% confidence interval (95%CIs) for prevalence data were calculated using multilevel logistic regression models to obtain robust standard errors that take into account the clustering by venue [42]. The hypothetically least stressful work conditions (i.e., low demands, high control, relaxed jobs, or stable) were selected as the reference categories in each indicator. Adjustments were made in steps in order to distinguish the different types of confounders. First: demographic characteristics; second: demographic characteristics and interpersonal factors; and third: all background covariates. For the purpose of identifying potential associations between psychosocial work conditions and smoking behavior, unless otherwise noted, respondents who reported ex-smokers were not included as the comparison group. All analyses were done with SAS software package version 9.03.

Given no comparable comparison groups could be found within the same workplace environment or extracted from the general population, the latest National Prevalence Survey of Smoking Pattern (NPSSP) was chosen as a comparison group.

## Results

### Demographic characteristics

The average age of the 2508 respondents included in the analyses, was 31.43 years old (SD = 8.56) with the range of 15 to 59. Mean working experience within hospitality settings was 7.32 years (SD = 5.63) with a range of 1 to 40 years.

### Patterns of smoking

**Figure 1:**
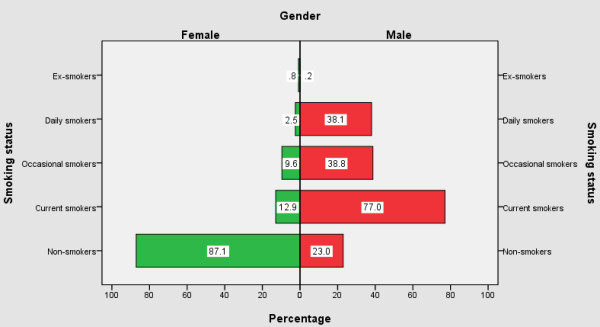
**Patterns of Smoking by Gender among 2508 Blue-collar and Service Workers Employed in Hospitality Venues in Shenyang, China, 2008 ^a^**. Note: ^a ^Current smokers: Ex-smokers, Occasional smokers, and Daily smokers combined.

Figure 1 presents the smoking status stratified by sex. Overall, the smoking prevalence for the study sample was 49.4%. Of which, 22.7 were identified as daily smokers, 26.2% were occasional smokers, and only 0.5% were ex-smokers. Significantly higher percentages were observed among male occasional smokers (38.8% *versus *9.7% (female); *p *< 0.001), and daily smokers (38.1% *versus *2.5% (female); *p *< 0.001). Table 1 shows the smoking rates by age group, with 52.4% of younger respondents (≤19 years) to be current smokers and one largest increase between ages 40 and 49.

**Table 1:** Patterns of Smoking by Age among 2508 Blue-collar and Service Workers Employed in Hospitality Venues in Shenyang, China, 2008^b^

Age, years	Never smokers (%)	Ever smokers (%)	Occasional smokers (%)	Daily smokers (%)	Current smokers (%)
≤19(n = 252)	47.6	0.0	34.9	17.5	52.4
20-29(n = 1031)	52.8	0.3	27.2	19.8	47.0
30-39(n = 796)	56.0	0.1	22.4	21.5	43.9
40-49(n = 363)	37.7	0.8	28.1	33.3	61.4
≥50(n = 66)	36.4	7.6	12.1	43.9	56.0

Note: ^b ^Current smokers are comprised of Occasional smokers and Daily smokers.

### Background characteristics by smoking status

Table 2 and Table 3 summarize the characteristics of the 2496 respondents (excluding 12 ex-smokers) stratified by smoking status. A total of 656 subjects (26.3%) reported to be occasional smokers. The significantly higher prevalence of them were younger (≤19 years), men, and lower-educated. On the other hand, the older (≥40 years), men, higher level (>3000 *yuan *RMB) family-income/month employees, lower-educated workers, and the divorced, separated, or widowed accounted for substantially higher proportion of daily smokers (*P *in all cases <0.05). Furthermore, those living with smoking parents constituted obviously higher percentage of both occasional and daily smokers, while both were evenly distributed between the groups stratified on the basis of peers' influence (positive or not).

**Table 2:** Demographic Characteristics by Smoking Status of 2508 Blue-collar and Service Workers Employed in Hospitality Venues in Shenyang, China, 2008^c^

Characteristics	N(%) of Never smokers	Occasional smokers		Daily smokers	
			
		N(%)	P value	N(%)	P value
*Overall*	1271(50.9)	656(26.3)		569(22.8)	
*Age(years)*					
≤19	120(47.6)	88(34.9)		44(17.5)	
20-29	544(52.9)	280(27.2)		204(19.8)	
30-39	446(56.1)	178(22.4)		171(21.5)	
40-49	137(38.1)	102(28.3)		121(33.6)	
≥50	24(39.3)	8(13.1)	P = 0.045	29(47.5)	P = 0.001
*Gender*					
Female	944(87.8)	104(9.7)		27(2.5)	
Male	327(23.0)	552(38.8)	P < 0.001	542(38.1)	P < 0.001
*Ethnicity*					
Han	1150(51.2)	575(25.6)		521(23.2)	
Others	121(48.4)	81(32.4)	P = 0.073	48(19.2)	P = 0.382
*Monthly family income*					
≤2000yuan	1153(51.5)	595(26.6)		491(21.9)	
2001-3000 yuan	73(45.6)	49(30.6)		38(23.8)	
3001-5000 yuan	35(53.8)	9(13.8)		21(32.4)	
≥5000yuan	10(31.3)	3(9.4)	P = 0.196	19(59.4)	P < 0.001
*Education*					
Junior school or lower	298(42.8)	214(30.7)		185(26.5)	
High and technical school	674(53.5)	315(25.0)		270(21.4)	
College or higher	299(55.4)	127(23.5)	P = 0.007	114(21.1)	P = 0.039
*Marital status*					
Married/Cohabiting	578(47.5)	370(30.4)		270(22.2)	
Single	668(55.2)	272(22.5)		270(22.3)	
Others^#^	25(36.8)	14(20.6)	P = 0.189	29(42.6)	P = 0.028

Notes: ^c^For the purpose of identifying potential associations between occupational correlates and smoking, respondents who reported Ex-smokers were not included (n = 12). The analyses were conducted separately for occasional smokers, and daily smokers compared with the non-smokers. ^# ^divorced, separated, and widowed combined.

**Table 3:** Other Background Characteristics by Smoking Status of 2508 Blue-collar and Service Workers Employed in Hospitality Venues in Shenyang, China, 2008^c^

Characteristics	N(%) of Never smokers	Occasional smokers		Daily smokers	
			
		N(%)	P value	N(%)	P value
*Interpersonal factors*					
*Paternal smoking*					
Yes	642(43.3)	440(29.7)		400(27.0)	
No	629(62.0)	216(21.3)	P < 0.001	169(16.7)	P < 0.001
*Maternal smoking*					
Yes	135(33.1)	155(38.0)		118(28.9)	
No	1136(54.4)	501(24.0)	P < 0.001	451(21.6)	P < 0.001
*More than one peers smoke*					
Yes	1174(50.7)	608(26.2)		535(23.1)	
No	97(54.2)	48(26.8)	P = 0.722	34(19.0)	P = 0.237
*Attitudinal factors of smoking*					
*An easy way to enjoy yourself*					
Disagree	517(57.8)	210(23.5)		168(18.8)	
Agree	754(47.1)	446(27.9)	P = 0.009	401(25.0)	P < 0.001

Notes: ^c^For the purpose of identifying potential associations between occupational correlates and smoking, respondents who reported Ex-smokers were not included (n = 12). The analyses were conducted separately for occasional smokers, and daily smokers compared with the non-smokers.

Table 4 shows that the ORs of occasionally smoking were significantly higher in men, among those living with smoking parents, and among employees with positive smoking-related attitude compared to the women, persons living without smoking parents, and persons with negative smoking-related attitude, respectively. Apart from characteristics that were common to occasionally smoking, the ORs of daily smoking were also significantly higher in the older age, among persons with high family-income, and among persons with unfortunate marriage than among the young (<40 years), persons with low family income, and single or married, respectively. Furthermore, adjustments for age and sex didn't alter these results. However, higher-educated employees were with significantly lower prevalence of both occasional smoking (OR 0.54, 95% CI 0.34 to 0.81) and daily smoking (OR 0.63, 95% CI 0.43 to 0.93).

**Table 4:** Logistic Regression Analysis of Background Characteristics across Smoking Status among 2508 Blue-collar and Service Workers Employed in Hospitality Venues in Shenyang, China, 2008 ^c, d^

Characteristics	Occasional smokers		Daily smokers	
		
	Crude OR(95%CI)	Adjusted OR(95%CI)	Crude OR(95%CI)	Adjusted OR(95%CI)
*Age(years)*				
≤19	1.00	1.00	1.00	1.00
20-29	0.64(0.31-1.33)	0.68(0.34-1.35)	1.01(0.65-1.57)	1.30(0.76-2.24)
30-39	0.43(0.22-0.85)	0.60(0.31-1.17)	1.08(0.57-2.07)	1.80(0.91-3.59)
40-49	0.85(0.47-1.56)	1.14(0.60-2.18)	2.50(1.30-4.80)	3.10(1.48-6.50)
≥50	0.35(0.12-0.97)	0.23(0.08-0.64)	3.51(1.42-8.70)	2.92(0.92-9.20)
*Gender*				
*Female*	1.00	1.00	1.00	1.00
*Male*	11.56(3.88-34.47)	11.19(3.77-33.24)	49.63(21.6-113.8)	49.55(21.9-112.3)
*Monthly family income*				
≤2000yuan	1.00	1.00	1.00	1.00
2001-3000 yuan	1.29(0.81-2.07)	1.51(0.90-2.53)	1.29(0.88-1.89)	1.21(0.73-2.00)
3001-5000 yuan	0.52(0.22-1.18)	0.79(0.30-2.08)	1.30(0.69-2.46)	1.78(0.89-3.57)
≥5000yuan	0.55(0.13-2.35)	0.32(0.07-1.39)	5.72(2.33-14.06)	8.22(1.20-56.12)
*Education*				
Junior school or lower	1.00	1.00	1.00	1.00
High and technical school	0.62(0.45-0.88)	0.69(0.44-1.08)	0.63(0.41-0.97)	0.96(0.53-1.74)
College or higher	0.54(0.34-0.87)	0.41(0.24-0.71)	0.63(0.43-0.93)	0.61(0.37-1.03)
*Marital status*				
Single	1.00	1.00	1.00	1.00
Married/cohabiting	0.73(0.47-1.13)	0.75(0.44-1.27)	1.19(0.75-1.91)	1.19(0.59-2.39)
Others	0.95(0.46-1.96)	0.89(0.42-1.88)	3.03(1.58-5.81)	2.74(1.02-8.14)
*Interpersonal factors*				
*Father's smoking*				
No	1.00	1.00	1.00	1.00
Yes	2.14(1.63-2.80)	2.54(1.86-3.47)	2.23(1.71-2.91)	2.75(1.96-3.86)
*Mother's smoking*				
No	1.00	1.00	1.00	1.00
Yes	2.64(1.89-3.69)	3.54(2.25-5.57)	2.19(1.60-2.99)	2.02(1.39-2.94)
*More than one peers smoke*				
No	1.00	1.00	1.00	1.00
Yes	1.07(0.74-1.55)	1.28(0.86-1.89)	1.27(0.85-1.88)	1.26(0.69-2.29)
*Attitudinal factors of smoking*				
*An easy way to enjoy yourself*				
Disagree	1.00	1.00	1.00	1.00
Agree	1.43(1.10-1.87)	1.42(1.11-1.82)	1.57(1.24-1.98)	1.60(1.20-2.13)

Notes: ^c^Respondents who reported Ex-smokers were not included (n = 12). The analyses were conducted separately for occasional smokers, and daily smokers compared with the non-smokers. ^d ^OR = odds ratio; CI = confidence interval; Adjusted OR: adjusted for age and gender.

### Psychosocial work conditions and smoking status

Table 5 shows that neither crude nor multivariate adjusted ORs suggested any significant relationship of occasionally smoking to job strain and any of its components. The ORs of occasionally smoking were significantly high in the "maybe stable" (adjusted OR 1.66, 95%CI 1.20 to 2.31) and "unstable" (adjusted OR 2.25, 95%CI 1.61 to 3.13) categories irrespective of adjustments for all background covariates compared to the reference stable job category.

**Table 5:** Associations between the Components of Occupational Correlates and Smoking Behavior in Occasional Smokers: Odds Ratios (ORs) and 95% Confidence Intervals (CIs) from Multilevel Logistic Regression Models among 2508 Blue-collar and Service Workers Employed in Hospitality Venues in Shenyang, China, 2008

Characteristics	N of participants	N(%) of Occasional smokers	Odds ratios (95% CI) for occasional smoking, adjusted for			
			
			None	A*	B*	C*
**Job demands**						
Quartile 1 (L)	859	220(33.5)	1.00	1.00	1.00	1.00
Quartile 2	455	113(17.2)	1.08(0.82-1.43)	1.22(0.93-1.59)	1.16(0.81-1.68)	1.17(0.81-1.69)
Quartile 3	669	189(28.8)	1.30(0.98-1.74)	1.27(0.90-1.78)	1.19(0.86-1.64)	1.18(0.86-1.63)
Quartile 4 (H)	513	134(20.4)	1.23(0.95-1.60)	1.41(0.97-2.05)	1.31(0.92-1.87)	1.32(0.93-1.88)
**Job control**						
Quartile 4 (H)	494	142(21.6)	1.00	1.00	1.00	1.00
Quartile 3	580	148(22.6)	1.11(0.87-1.42)	0.99(0.75-1.31)	0.90(0.62-1.31)	0.91(0.62-1.32)
Quartile 2	626	168(25.6)	1.14(0.89-1.46)	1.05(0.77-1.44)	0.92(0.64-1.33)	0.93(0.65-1.34)
Quartile 1 (L)	796	198(30.2)	1.00(0.76-1.31)	1.16(0.83-1.61)	1.03(0.73-1.47)	1.02(0.72-1.48)
**Job strain**						
Relaxed jobs	455	121(18.4)	1.00	1.00	1.00	1.00
Active jobs	575	169(25.8)	1.19(0.94-1.50)	0.99(0.71-1.38)	1.02(0.70-1.49)	1.02(0.69-1.50)
Passive jobs	906	212(32.3)	0.98(0.72-1.34)	0.78(0.55-1.09)	0.92(0.64-1.33)	0.93(0.64-1.34)
Strain jobs	560	154(23.5)	1.27(0.93-1.74)	1.14(0.79-1.66)	1.26(0.58-1.87)	1.26(0.84-1.88)
**Job stability**						
Stable	615	146(22.3)	1.00	1.00	1.00	1.00
Maybe stable	952	240(36.6)	1.22(0.95-1.56)	1.65(1.24-2.18)	1.67(1.20-2.30)	1.66(1.20-2.31)
Unstable	929	270(41.1)	1.70(1.22-2.37)	2.40(1.70-3.37)	2.25(1.62-3.12)	2.25(1.61-3.13)

Notes: A* odds ratios are adjusted for all demographic characteristics; B* in addition to demographic characteristics, odds ratios are adjusted for the interpersonal factors; C* odds ratios are adjusted for all background covariates; L = low; H = high.

Table 6 depicts the results from multilevel logistic regression analyses on the associations between psychosocial work conditions and daily smoking status. Of which, strain jobs (OR 1.88, 95%CI 1.28 to 2.77) and passive jobs (OR 1.97, 95%CI 1.38 to 2.82) were significantly associated with an increased likelihood of daily smoking. Of the components of the job strain model, low job control (OR 3.31, 95%CI 2.24 to 4.89) and high job demands (OR 1.44, 95%CI 1.03 to 2.13) were also associated with higher odds for smoking daily. Furthermore, ORs in the strain jobs (adjusted OR 2.09, 95%CI 1.28 to 3.41), passive jobs (adjusted OR 2.01, 95%CI 1.27 to 3.17), low job control (adjusted OR 2.56, 95%CI 1.57 to 4.16), and high job demands (adjusted OR 1.72, 95%CI 1.13 to 2.61) categories were only weakly affected by the adjustments in the multivariate analyses. Similar as occasionally smoking, employees with "maybe stable" and "unstable" jobs had significantly high ORs of daily smoking.

**Table 6:** Associations between the Components of Occupational Correlates and Smoking Behavior in Daily Smokers: Odds Ratios (ORs) and 95% Confidence Intervals (CIs) from Multilevel Logistic Regression Models among 2508 Blue-collar and Service Workers Employed in Hospitality Venues in Shenyang, China, 2008

Characteristics	N of participants	N(%) of Daily smokers	Odds ratios (95% CI) for daily smoking, adjusted for			
			
			None	A*	B*	C*
**Job demands**						
Quartile 1 (L)	859	152(26.7)	1.00	1.00	1.00	1.00
Quartile 2	455	103(18.1)	1.31(0.93-1.85)	1.65(1.11-2.45)	1.70(1.10-2.64)	1.66(1.07-2.57)
Quartile 3	669	174(30.6)	1.38(1.02-1.86)	1.60(1.14-2.26)	1.55(1.06-2.27)	1.53(1.04-2.24)
Quartile 4 (H)	513	140(24.6)	1.44(1.03-2.13)	1.71(1.17-2.50)	1.76(1.16-2.67)	1.72(1.13-2.61)
**Job control**						
Quartile 4 (H)	494	55(9.7)	1.00	1.00	1.00	1.00
Quartile 3	580	163(28.6)	2.61(1.79-3.80)	1.93(1.23-3.03)	2.11(1.32-3.37)	1.97(1.24-3.15)
Quartile 2	626	146(25.7)	2.24(1.52-3.32)	1.63(1.02-2.61)	1.69(1.04-2.74)	1.54(0.95-2.50)
Quartile 1 (L)	796	205(36.0)	3.31(2.24-4.89)	2.82(1.76-4.52)	2.77(1.71-4.50)	2.56(1.57-4.16)
**Job strain**						
Relaxed jobs	455	66(11.6)	1.00	1.00	1.00	1.00
Active jobs	575	108(19.0)	1.19(0.80-1.77)	1.25(0.78-2.02)	1.32(0.80-2.17)	1.31(0.81-2.16)
Passive jobs	906	236(41.5)	1.97(1.38-2.82)	1.68(1.09-2.60)	2.10(1.33-3.32)	2.01(1.27-3.17)
Strain jobs	560	159(27.9)	1.88(1.28-2.77)	1.78(1.11-2.84)	2.11(1.29-3.44)	2.09(1.28-3.41)
**Job stability**						
Stable	615	95(15.4)	1.00	1.00	1.00	1.00
Maybe stable	952	212(37.4)	1.77(1.29-2.43)	2.59(1.77-3.79)	2.58(1.73-3.83)	2.55(1.71-3.79)
Unstable	929	232(46.2)	2.59(1.89-3.54)	3.65(2.50-5.35)	3.40(2.29-5.05)	3.38(2.27-5.04)

Notes: A* odds ratios are adjusted for all demographic characteristics; B* in addition to demographic characteristics, odds ratios are adjusted for the interpersonal factors; C* odds ratios are adjusted for all background covariates; L = low; H = high.

## Discussion

There are four main results arising from present cross-sectional study among Shenyang hospitality industry blue-collar and service employees. First, hospitality industry blue-collar and service employees smoked at a rate 1.4 times that of the people nation-wide (49.4% vs. 35.8%), with 1.2-fold (77.4% vs. 66.0%) for male employees and 4.2-fold (12.9% vs. 3.08%) for female employees [43]. Furthermore, young employees (≤19 years) smoked at a rate 1.8 times that of the general population of comparable ages (52.4% vs. 29.3%) [44-46]. It should be noted that the above data were obtained by interviews in community surveys and may be not comparable with our data which were conducted specially in the hospitality industry workers. Nevertheless, these data show clearly that smoking in blue-collar and service employees is certainly a major public and occupational health problem in China.

Second, present study confirmed several socioeconomic predictors of smoking such as gender, education, marital status, and parents' smoking [47-49]. However, the findings also demonstrated two interesting conclusions: (1) blue-collar and service employees' occasional smoking behavior seem to be more influenced by their maternal smoking (OR 3.54,95% CI 2.25 to 5.57), compared with paternal smoking (OR 2.54,95%CI 1.86 to 3.47). This is probably because in this study occasional smokers comprised of more female (15.9%) than daily smokers (4.7%). Girls usually see their mothers as role models, and will emulate many habits prevalent in their mothers, including smoking evidently [50]. In addition, maternal supervision was found to be significant protective factors for smoking in these young girls [51,52]. For mothers, it will also be more difficult to stop their daughters from smoking if they themselves are smokers. (2) *Perrine et al. *[53] found that the risk was 4 times higher if one friend was a smoker and increased up to 160 times higher if 4 of the friends were smokers. However, in present study, neither the occasional smoking nor the daily smoking was significantly associated with peers' pressure. Perhaps not surprisingly, China is a traditional collective society where the family unit is more important than an individual or their peers. The influence of the peer smoking may depend on the type of society under studied (collective versus individualistic).

Third, neither job strain nor any of its components was significantly associated with occasional smoking status. This may be because smoking is usually started before entering into full time work--in adolescence. Nevertheless, smoking frequency and intensity can vary under workplace stress influences. In accordance with this hypothesis, there is more evidence for an association between job strain and daily smoking than for job strain and non-daily smoking [54,55]. We would, therefore, prefer to interpret the observed associations in terms of the decision to continue or enhance rather than to start smoking.

Finally, strain jobs, passive jobs, low job control, and high job demands were significantly and positively associated with daily smoking. Demographic characteristics, interpersonal factors, and attitude to smoking could be confounders of the associations between the job strain variables and daily smoking. Adjusting for these variables, however, only marginally affected the estimates. In general, our findings are consistent with studies suggesting an association of job strain [56,57], low job control [9,22], and high job demands [20,26] with smoking behavior. However, other studies have reported no association between job strain variables and smoking behavior [22,25,24,58,59]. Potential reasons for these inconsistencies include (a) predominantly covered male populations in earlier studies; (b) differences in measurement of control and demands across studies; and (c) smoking habits and the intensity of job strain differ in different professions. The result of passive jobs (i.e. low demand combined with low control) to be predictive of daily smoking was not initially hypothesized, although there is previous evidence of passive work conditions being hazardous to health [40]. It may be speculated that passive jobs are experienced as unimportant or worthless jobs not giving any occupational satisfaction [60] and in this sense they may increase intentions to and intensity of smoking.

There is a growing body of evidence showing that unstable employment is associated with health-related behaviors of smoking, alcohol usage, and physical inactivity [61-63]. However, earlier studies are inconsistent on the possible relationship between job unstability and health-related behavior; moreover, for none of these results is previous evidence as yet considered to be convincing. Findings from present study were consistent with our hypothesis that unstable employment were significantly associated with smoking of both occasional (OR 2.25, 95% CI 1.61 to 3.13) and daily (OR 3.38, 95% CI 2.27 to 5.04), irrespective of adjustments for all background covariates.

However, our study is subject to several limitations. Firstly, the reliance on cross sectional self-reported data in this study may have been influenced by recall bias (information bias). Secondly, self report data on substance use are often subject to underreporting. However, these may lead to underestimation rather than overestimation of the actual associations. Clearly, on the other hand, future longitudinal studies using both self reported and objective indicators of smoking (e.g., serum cotinine levels) and job strain variables would provide interesting comparisons to these findings.

Thirdly, the Chinese versions of the job control and job demands measures were derived from the Job Content Questionnaire [37], a valid and reliable tool for measuring occupational stress, but it remains to be seen among Chinese blue-collar and service employees within hospitality venues. Moreover, it did not include the measure of social support. It is possible that this has reduced the validity of the job strain assessments.

Fourthly, although we took several confounding factors into account, there are other potential factors that we were not able to in the analyses. For example, instrumental and emotional supports from colleagues, friends, or families, might influence the associations between job strain variables and smoking behavior [19]. Other unmeasured factors possibly related to smoking, such as alcohol consumption [64], long working hours, shift work, and the frequency of job problems [17] could also confound the results if they are also related to job strain variables.

Fifthly, applications of smoking restrictions varied between different work places. Although comprehensive smoke free policies had been implemented from all employers' statements, in fact, smoke-free laws had been exempted in various kinds of hospitality venues, with only 32.1% of the employees reporting good enforcement of smoke-free laws (unpublished data). There is evidence showing an association between workplace smoking restriction policy and employees' smoking behavior [65]. In addition, the respondents were blue-collar and service employees selected from Shenyang hospitality venues. The extent to which our results can be generalized to other populations is not known and the findings should be interpreted with caution until they are validated in studies using other samples.

Finally, present study used a cluster sampling design by venue. Given that the variables may show cluster effects (individuals at a venue having more similar experiences), many methodological challenges of cluster randomization arise because inferences are usually intended to apply at the individual level, while randomization is at the cluster level. Application of traditional statistical methods, which invariably assume no between cluster variation, will tend to bias observed *p *values underestimated and confidence intervals to narrow, thus risking a spurious claim of statistical significance and producing an artificially precise estimate of the association between psychosocial work characteristics and smoking status [66]. However, no significant differences in results were observed when taking clustering into account or not. Maybe because of higher turnover and shorter working period in the same venues among blue-collar and service employees in China, the cluster effects are sufficient small as to be ignorable (at the venues level, ICCs were all below 0.01 and corresponding design effects were in the range of 1.00-1.11) [67-69].

## Conclusions

Present study revealed that hospitality blue-collar and service employees in Shenyang, China, particularly female employees, smoke at much higher rates than the general population, and are much less likely to be ex-smokers. Our findings also lent some support to the notion that psychosocial work conditions defined as job strain and its components, and job stability were significantly related to daily smoking status, which, in a sense, extended knowledge on the potential indirect pathways (unhealthy behaviors) through which psychosocial work conditions may affect health.

## Practical implications

According to the findings from present study, urgent tobacco control measures are needed to prevent the epidemic of smoking among blue-collar and service employees under hospitality settings in Shenyang. As stressful job characteristics are potentially modified, if causal, the observed associations can have important implications for smoking prevention and quitting. First, our findings suggest that reducing stress by increasing job control, decreasing job strain, decreasing job demands, as well as increasing job stability might help the smoking cessation efforts. Furthermore, studies of smoking interventions combined with interventions targeted to reduce job strain would be a step forward in testing the hypothesis that integrated job stress and smoking intervention strategies should produce greater smoking cessation rates than traditional smoking intervention alone.

## List of abbreviations

OR: Odds Ratio; CI: Confidence Interval; RMB: Ren Min Bi (¥); SHIES: Shenyang Hospitality Industry Employees Survey; NPSSP: National Prevalence Survey of Smoking Pattern.

## Competing interests

The authors declare that they have no competing interests.

## Authors' contributions

XL, HYL, and BSZ were responsible for the overall design of the study and designed the survey strategy; XL, HYL, and XLL conducted the surveys and retrieved the questionnaires; XL, HYL, and XLL were responsible for the statistical analyses; XL, HYL, XLL, PG, ZHY, and BSZ have attended developing the article. All authors have read and approved the final manuscript.

## Pre-publication history

The pre-publication history for this paper can be accessed here:

http://www.biomedcentral.com/1471-2458/10/37/prepub
